# Temporal Patterning of Neurofilament Light as a Blood-Based Biomarker for Stroke: A Systematic Review and Meta-Analysis

**DOI:** 10.3389/fneur.2022.841898

**Published:** 2022-05-16

**Authors:** Jasmin D. Sanchez, Richard A. Martirosian, Katherine T. Mun, Davis S. Chong, Irene Lorenzo Llorente, Timo Uphaus, Klaus Gröschel, Teresa A. Wölfer, Steffen Tiedt, Jason D. Hinman, the DEMDAS Study Group

**Affiliations:** ^1^Indiana University School of Medicine, Indianapolis, IN, United States; ^2^University of Arizona College of Medicine-Tucson, Tucson, AZ, United States; ^3^David Geffen School of Medicine, University of California, Los Angeles, Los Angeles, CA, United States; ^4^Department of Neurology, University Medical Center of the Johannes Gutenberg University Mainz, Mainz, Germany; ^5^Institute for Stroke and Dementia Research, University Hospital, Ludwig-Maximilians-Universitat (LMU) Munich, Munich, Germany

**Keywords:** stroke, neurofilament (NF), biomarker, meta-analysis, cerebrovascular disease

## Abstract

Damage to axons is a core feature of ischemic stroke and cerebrovascular disease. The burden of axonal injury is correlated with the acute clinical deficits, the underlying burden of ischemic brain injury, the prognosis of recovery, and may be a meaningful therapeutic target for brain repair. Neurofilament light chain (NfL) has been identified as a blood-based biomarker that reflects neuroaxonal damage resulting from stroke. However, the utility of NfL as a blood-based biomarker in stroke is confounded by studies examining different temporal windows and patient populations. We conducted a systematic review and meta-analysis to verify the utility of blood NfL as a diagnostic, prognostic, and monitoring stroke biomarker. Nineteen studies reporting serum/plasma NfL values for a total of 4,237 distinct patients with stroke were identified. Using available summary data from the 10 studies that employed a common immunoassay platform, we utilized random effects linear mixed modeling and weighted averages to create a phasic model of serum/plasma NfL values in distinct time periods of acute stroke. Weighted averages show that blood NfL levels vary significantly across three distinct temporal epochs of acute (0–7 days), subacute (9–90 days), and chronic (>90 days) stroke with a steep peak in the early subacute period between 14 and 21 days after stroke. Blood NfL values can function as a diagnostic biomarker in distinguishing acute ischemic stroke from transient ischemic attack as well as amongst other cerebrovascular subtypes. Release of NfL into the bloodstream after stroke follows a distinct temporal dynamic that lags several weeks behind stroke onset and reliably associates with a stroke diagnosis despite some variability based on stroke subtype and severity. Identification of these temporal dynamics and the contribution of co- existent cerebrovascular disease states can improve the value of NfL as a stroke biomarker.

## Introduction

Defining the distinct physiological stages of stroke using biomarkers is critical to the development of treatments and interventions for time points beyond the hyperacute period of stroke. In recent years, with the emergence of ultrasensitive blood tests detecting neurofilament light chain (NfL), blood NfL has been identified as a sensitive but non-specific marker of neuro-axonal injury (1, 2). As an essential component of the neuronal cytoskeleton and the structural backbone of neuronal connectivity, NfL is uniquely positioned to function as a surrogate for neurologic disease (3). Highly sensitive biomarkers with low specificity, such as NfL, may have limited roles in neurologic diagnosis without context but harbor the potential to be extremely useful in paradigms of disease staging, prognosis, determination of therapeutic candidacy, and assessment of response to reparative interventions. Stroke is a particularly excellent representative model upon which to layer blood-based biomarkers as they can potentially assist in addressing existing and emerging clinical challenges in stroke care. In stroke, NfL is an attractive target as a biomarker because the axon is rapidly susceptible to ischemic injury (4), undergoes a progressive breakdown after ischemia, and the extent of axon damage is strongly tied to functional recovery (5). Thus, precise and accurate measurements of NfL levels after stroke can assist in identifying patterns of recovery and clearly delineating therapeutic windows that may exist for therapeutic interventions including delayed revascularization therapies (6), axonal outgrowth therapies (7), stem cell treatments, and rehabilitative therapies (8).

Given the universality of axonal injury resulting from stroke in both animal models and the clinical setting, NfL is also uniquely positioned to function as a translational biomarker. However, widespread adoption of a reliable biomarker requires consistent reproducibility across labs and clinical settings (9). Measurement of NfL on different assay platforms and in varying clinical cohorts with cerebrovascular disease has created confusion about the true absolute detection of blood NfL levels and its variance in post-stroke periods. Measurements of blood NfL levels after stroke have included post-stroke epochs ranging from time of hospitalization to as far out as 450 days post-stroke with inconsistent time intervals across studies. Together these issues have limited the utility of blood NfL despite its potential as an ideal post-stroke biomarker with potential application in clinical trials.

In this meta-analysis, we gathered all available studies measuring blood NfL values in clinical acute stroke and chronic cerebrovascular disease states. We used this pooled data to create a model of temporal variations in measured blood NfL levels after stroke relative to control subjects and chronic cerebrovascular disease states. In addition, we assessed the ability of blood NfL values to determine the likelihood of a stroke diagnosis and reviewed the literature available for stroke outcome prognosis. The utility of such a detailed model would not only clarify the role of blood NfL in determining post-stroke injury trajectories but would also provide a template for the use of blood NfL as a translational biomarker for emerging post-stroke reparative therapeutics. We achieved these goals and developed a web-based interface allowing subject level queries to estimate the likelihood of a stroke diagnosis and determine the most likely post-stroke window using measured blood NfL values.

## Methods

### Literature Search Strategy

The overall goal of this study was to capture all articles that related to peripheral blood detection of neurofilament light in research subjects with a diagnosis of acute ischemic stroke or chronic cerebrovascular disease including cerebral small vessel disease and cerebral autosomal dominant arteriopathy with subcortical infarcts and leukoencephalopathy (CADASIL). The search strategy was developed to identify all available articles reporting plasma or serum NfL values in relationship to stroke and cerebrovascular disease. Literature searches were conducted between July 1^st^ and November 15^th^ 2020 using the NIH National Library of Medicine PubMed Database and the EMBASE biomedical research database. The following search terms were used for both database searches: “neurofilament light stroke,” “neurofilament light stroke serum,” “neurofilament light plasma injury,” and “neurofilament light stroke animal.” There were no language limitations placed on the searches conducted. Inclusion criteria included human subjects with a diagnosis of stroke or qualifying chronic cerebrovascular disease and reportable median and standard deviation of blood NfL values. Identified articles were excluded if they reported non-original research or did not report numerical values for blood NfL in tabular or text format.

### Assessment of Bias

Identified articles were reviewed independently by two authors (KM, JDH) for potential impact of bias using the QUADAS-2 method (10).

### Data Extraction

Data extraction was performed by two authors (JS, RM) and data verification and conflict resolution performed by two others (KM, JDH). Using available summary data (median, IQR) from the text or tables provided, a database of median NfL values organized by time after stroke and in other cerebrovascular conditions was created. Extracted data by article is provided as Supplemental Information.

### Statistical Modeling

Eight studies examining serum/plasma NfL variation after stroke were used in a random effects linear mixed model to determine the effect of time windows in acute stroke assuming statistical significance at *t* <2 using the *t-z* method (11). Estimated distributions of NfL values for control, acute (0–7 days), subacute (9–90 days), and chronic (> 90 days) time windows after stroke were used to calculate normal distributions and probability matrices for the likelihood of stroke and likelihood of a particular time window after stroke for any given NfL value between a range of 5–250 pg/ml based on the Bayes rule.

In addition, we used all studies employing the SIMOA (Quanterix) platform to create a weighted average (median NfL value^*^study *n*)/(total studies *n*) for individual time points after stroke and for cerebrovascular subtypes. Aggregate IQR ranges using the identical formula substituting upper and lower IQR values for median NfL value. When summary data was presented across a time range (e.g. 0–8 days), we used the median time point to represent the summary data from that study as a single time point. Comparisons between varying cerebrovascular subtypes were done by treating median = mean and using a one-way ANOVA and Tukey's correction for multiple comparisons assuming adjusted *p* < 0.05. Analysis and graphing performed using Microsoft Excel, SPSS, and Prism.

## Results

### Evidence for Blood NfL as a Biomarker for Stroke and Cerebrovascular Disease

**Figure 1:**
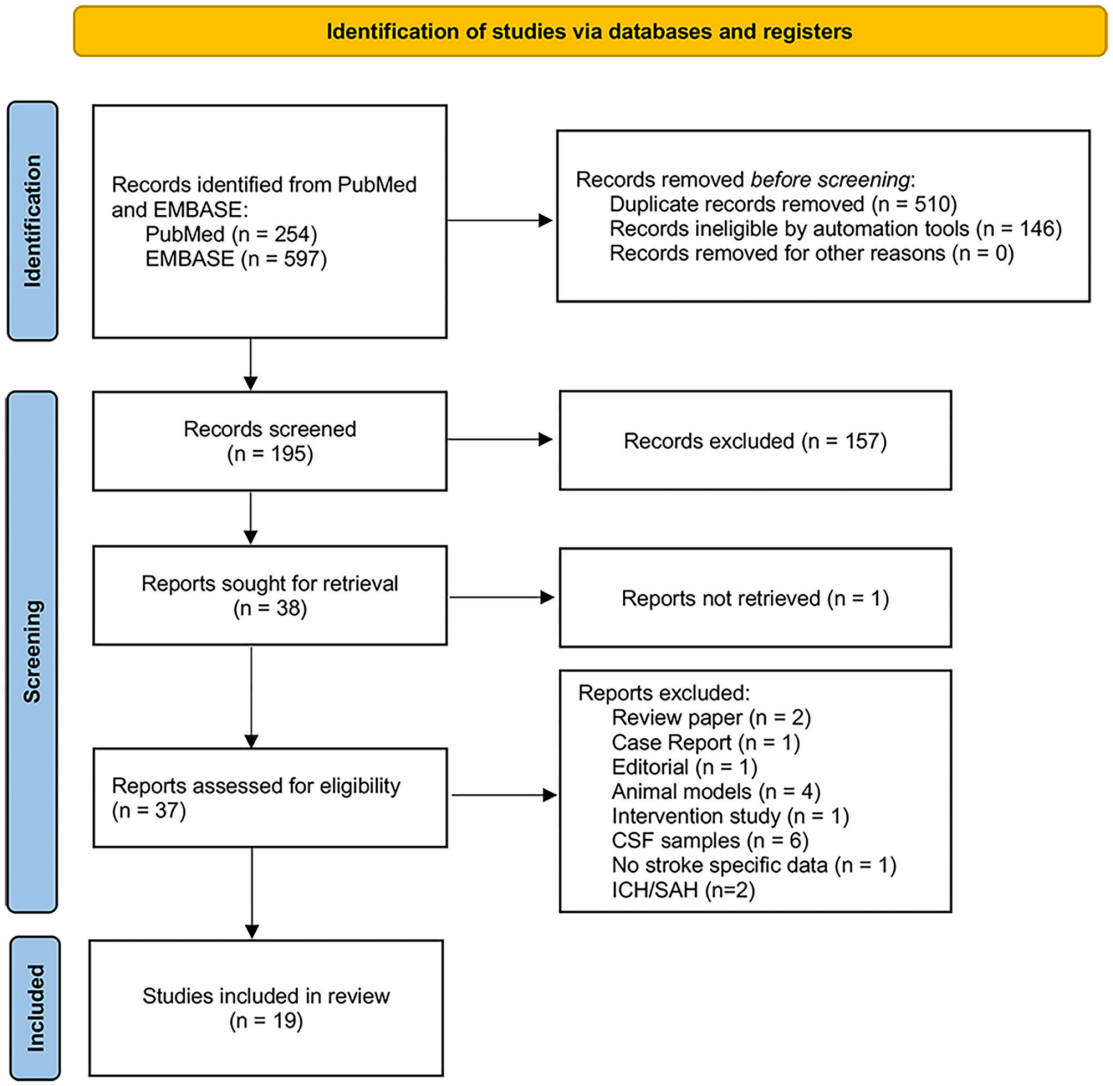
PRISMA flow diagram showing search and study selection process.

Using the search strategy detailed in Figure 1, we identified a total of 851 records (PubMed = 254, EMBASE = 597) and after removal of duplicates and non-original research, there were 195 distinct records. Screening resulting in 38 articles sought for retrieval, with one record unavailable for retrieval. Of the 37 reports assessed, 19 articles reported serum or plasma NfL measurements for stroke and cerebrovascular disease. These 19 articles were qualitatively reviewed and a subset of 16 articles, which used the ultrasensitive single molecule array (SIMOA) platform for NfL measurement, were used in the final quantitative analysis.

Nineteen articles contained serum or plasma values for NfL in patients with stroke or cerebrovascular disease (Table 1). We observed no obvious differences between studies employing plasma or serum detection of NfL levels. For simplicity throughout the remainder of the text, we refer to NfL levels as derived from blood. Details on the precise source of fluid are available in Table 1.

**Table 1:** Articles included in meta-analysis of blood NfL as stroke and cerebrovascular disease biomarker.

**Publication (PMID)**	**Subjects**	**Blood specimen**	**Stroke subtypes**	**Time period of measurement**	**Median baseline NIHSS (IQR)**	**Median outcome mRS (IQR)**	**Included in RELMM**
Gattringer et al. (12) (29046363)	79	Serum	RSSI	Baseline, D90, 15mo	n/a	n/a	Yes
Pinter et al. (13) (29737904)	78	Serum	RSSI	Baseline, D90, 15mo	Baseline: 2.0 (3.0) D90: 0.0 (1.0) 15mo: 0.0 (1.0)	n/a	No
Duering et al. (14) (29886723)	CADASIL: 53 CSVD: 439	Serum	CADASIL, CSVD	D1	0 (1)	n/a	No
Tiedt et al. (15) (30217937)	196 (CIRCULAS) 95 (DEMDAS)	Serum	AIS	D1, D2, D3, D7, D90, 6mo	2 (4)	1 (1–2)	Yes
Gravesteijn et al. (16) (30656183)	41	Serum	CADASIL	Baseline, 7 yr	n/a	n/a	No
Pujol-Calderón et al. (17) (30599262)	30	Serum	AIS	D1, D2-3, D7-9, D90	n/a	n/a	Yes
Korley et al. (18) (31138085)	113	Serum	CSVD	Baseline	n/a	n/a	No
Onatsu et al. (19) (31151840)	AIS: 101 TIA: 35	Serum	AIS, TIA	Baseline, D1, prior stroke	3.7	0.33+/-0.82	Yes
Pedersen et al. (20) (31375988)	595	Serum	AIS	Baseline, D90, 7 yr	2.9 (1.6–7.2)	D90: 2 (1–2) 7yr: 2 (1–4)	Yes
Uphaus et al. (21) (31537188)	211	Serum	AIS	D1, 12mo	4 (2–8)	>/ = 2	Yes
Wang et al. (22) (31898161)	343	Serum	AIS	Baseline	6 (4–11)	1 (0–2)	Yes
Zhao et al. (23) (32078857)	AIS: 236 TIA: 80	Serum	AIS, TIA	Baseline, D1, D3, D7, D14, D21, D28	n/a	n/a	Yes
Chen et al. (24) (32321529)	63	Plasma	CADASIL	Baseline	n/a	n/a	No
Nielsen et al. (25) (32595585)	AIS: 31 TIA: 9	Serum	AIS, TIA	Baseline, D3	AIS: 3.3 (1.8–5.7)* TIA: 0.7 (0.7–4.2)*	AIS: 1 (1–3)** TIA: 0 (0)	Yes
Peters et al. (26) (33053952)	503	Serum	CSVD	Baseline	n/a	n/a	No
Gendron et al. (27) (33177179)	227	Plasma	AIS	D0-D8, D9-D20	4 (0–37)	3 (2–4)	Yes
Traenka et al. (28) (26418549)	AIS: 31 TIA: 10	Serum	AIS, TIA	D0-D30	4 (0.5–9.75)	n/a	No
De Marchis et al. (29) (29281157)	AIS: 504 TIA: 111	Serum	AIS, TIA	Baseline, D1, D2, D90	AIS: 5 (2–10) TIA: 0 (0–2)	n/a	No
O'Connell et al. (30) (32353434)	14	Plasma	AIS	D1	6.3 +/-7.8 (SD)	n/a	No

RSSI, recent small subcortical infarct; CADASIL, cerebral autosomal dominant arteriopathy with subacute infarcts and leukoencephalopathy; CSVD, cerebral small vessel disease; AIS, acute ischemic stroke; TIA, transient ischemic stroke; IS, ischemic stroke; RELMM, Random effects linear mixed model. Gray shading: Blood NfL level measured on SIMOA platform. White shading: Blood NfL level measured using other immunoassay platform. ^*^Converted NIHSS from Scandinavian Stroke Scale (SSS) using formula: NIHSS = 25.68–0.43(SSS). Data for two AIS participants missing from calculation. ^**^Data missing from four participants.

Ten articles measured blood NfL values over time in 2,268 patients with acute cerebrovascular events (acute ischemic stroke, transient ischemic attack, or recent small subcortical infarct) and in 827 control subjects (12, 15, 17, 19–23, 25, 27). One additional article examined acute cerebrovascular event NfL levels on a previously reported cohort (13). Five articles measured blood NfL levels in patients with cerebral small vessel disease (CSVD) or cerebral autosomal dominant arteriopathy with subacute infarcts and leukoencephalopathy (CADASIL) (14, 16, 18, 24, 26). These 16 articles measured blood NfL values using the ultrasensitive SIMOA platform (Table 1). The remaining three articles measured blood NfL values using a locally developed electrochemiluminescence immunoassay (ECLIA) (28, 31), a digital ELISA (30), or a ELISA- ECL assay (29, 32). The included studies examined a wide variety of stroke subtypes, temporal windows, and outcome measures. Assessment of bias did not reveal any significant influence on included study results (Supplementary Figure 1). In the subsequent sections, we summarize the clinical populations and critically assess the various comparisons utilized in these studies. Median and interquartile range data are included for each study whenever available.

### Value of Blood NfL as a Predictor of an Acute Stroke Diagnosis

Seven articles focused on the predictive ability of blood NfL to discriminate acute ischemic stroke (AIS) subjects from control or transient ischemic attack (TIA) subjects (12, 15, 19, 25, 28–30). Traenka et al. measured baseline serum NfL levels in a cohort of patients (*n* = 49) presenting with cervical artery dissection and AIS [46.3 pg/ml (20.5–171.1)] in addition to those patients experiencing only local symptoms (Horner's syndrome, tinnitus or cranial nerve palsy) or TIA. Patients with cervical artery dissections presenting with AIS had significantly higher serum NfL levels than patients with TIA or local symptoms [108.9 pg/ml (37.8–427.7), 16.4 pg/ml (8.7–36.3), 23.4 pg/ml (17.8–30.8)]. Three studies examined blood NfL values in prospective cohorts of patients presenting with stroke or TIA (19, 25, 29). De Marchis et al. measured serum NfL upon admission in 504 AIS patients and 111 TIA patients and found a significant difference between AIS and TIA [16 pg/ml (7–34) vs. 9 pg/ml (4–19)]. Onatsu et al. included 136 stroke and TIA patients in a similar prospective cohort study measuring serum NfL using the SIMOA platform upon hospital admission. They found a significant difference in NfL values between AIS vs. TIA patients [89.5 pg/ml (44.7–195.3) vs. 25.2 pg/ml (14.6–48.0)] that remained after adjustment for age, NIHSS, and stroke volume. These authors proposed a cutoff value of 49.0 pg/ml for a stroke diagnosis (sensitivity of 73%; specificity 80%). Nielson et al. found a significant difference in plasma NfL in patients with acute ischemic stroke at <8 h from stroke onset [28.70 pg/ml (15.20–68.30) and 72 h from stroke onset [31.70 pg/ml (16.70–98.40)] compared to healthy controls [14.10 pg/ml (7.73–18.96)], but no discriminatory value compared to TIA patients [20.90 pg/ml (9.30–41.25)] in a small cohort. The remaining three articles included convenience control cohorts totaling 116 as comparators to AIS patients and found consistent elevations of blood NfL levels in AIS. Notable in these studies are the significant variance between studies in the absolute detection level of NfL associated with a stroke diagnosis: 16 pg/ml vs. 49 pg/ml. This could be due to differences in populations and/or technical differences in assay protocols.

### Association of Blood NfL Values With Stroke Severity

Nine studies evaluated the association of between stroke severity and axonal damage measured by blood NfL levels (15, 19–22, 25, 27–30). In studies including 2,405 subjects, both clinical stroke severity at onset and radiographic infarct volumes appears to be associated with blood NfL levels. The data is strongest for clinical severity measured by National Institute of Health Stroke Scale (NIHSS) while the association with radiographic indicators is largely dependent on when infarct volumes are measured, and which imaging modality is examined. Infarcts visible on admission CT are much more likely to be associated with an increased blood NfL value while the highly sensitive diffusion-weighted MR imaging technique correlates tightly once final infarct volumes are established in the late acute period.

In an early small-scale study, Traenka et al. showed that serum NfL levels associated with NIHSS with a weak linear relationship reported. Wang et al. measured serum NfL at baseline in 343 AIS patients and found that NfL values associated with infarct volume by imaging analysis. Serum NfL concentration in patients with a moderate to high stroke severity (NIHSS ≥5) were higher than that observed in patients with minor clinical severity [21.2 pg/ml (15.1–31.7) vs. 14.9 pg/ml (11.8–19.4)]. Uphaus et al. showed a significant correlation between NIHSS and serum NfL measured 24 h after hospital admission. In subjects with NIHSS >4, serum NfL values were 22.9% higher. Pedersen et al. found a significant association between baseline serum NfL values and initial NIHSS. In this study, the median NIHSS was 2.9 (1.6–7.2) yet the median serum NfL value in cases was increased more than 4-fold compared to controls [60.2 pg/ml (28.3–109.4) vs. 14.2 pg/ml (9.7–21), respectively]. Nielsen et al. found serum NfL values associated with stroke severity as measured by the Scandinavian Stroke Scale. In the largest single study of this type, De Marchis et al. established median serum NfL values associated with specific NIHSS severity subgroups [NIHSS <7 = 13.1 pg/ml (5.3–27.8); NIHSS 7–15 = 16.7 pg/ml (7.4–34.9); NIHSS >15 = 21.0 pg/ml (9.3–40.4)]. When comparing patients with similar NIHSS, serum NfL values were higher in those with AIS compared to TIA. These authors did not find an association with serum NfL and infarct size as measured on admission MRI.

Gendron et al. demonstrated both an association of plasma NfL levels with stroke severity as judged by admission NIHSS as well as a radiographic association with the Alberta Stroke Program Early Computerized Tomography Score (ASPECTS). In this study, every doubling of plasma NfL was associated with a 55% increased chance of a lower ASPECTS, consistent with a larger and more severe stroke, though the plasma draw and imaging were not time-locked. Onatsu et al. also showed an association between serum NfL and stroke severity by initial NIHSS (mean 3.7) as well as a fairly linear relationship between radiographic measure of infarct volume measured in the subacute period. Notably, these authors documented a significant difference in baseline serum NfL values between stroke subjects with an infarct visible on CT and those without [87.3 pg/ml (46.1–187.2) vs. 40.9 pg/ml (18.3–83.9), respectively]. Tiedt et al. showed that serum NfL values strongly correlated with infarct volume by MRI measured at the day 7 post-AIS peak serum NfL level observed in this study. In a small-scale study, using digital ELISA, O'Connell et al. demonstrated that plasma NfL was associated with infarct volume on CT/MRI.

### Temporal Variation in Blood NfL Values During and After Stroke

**Figure 2:**
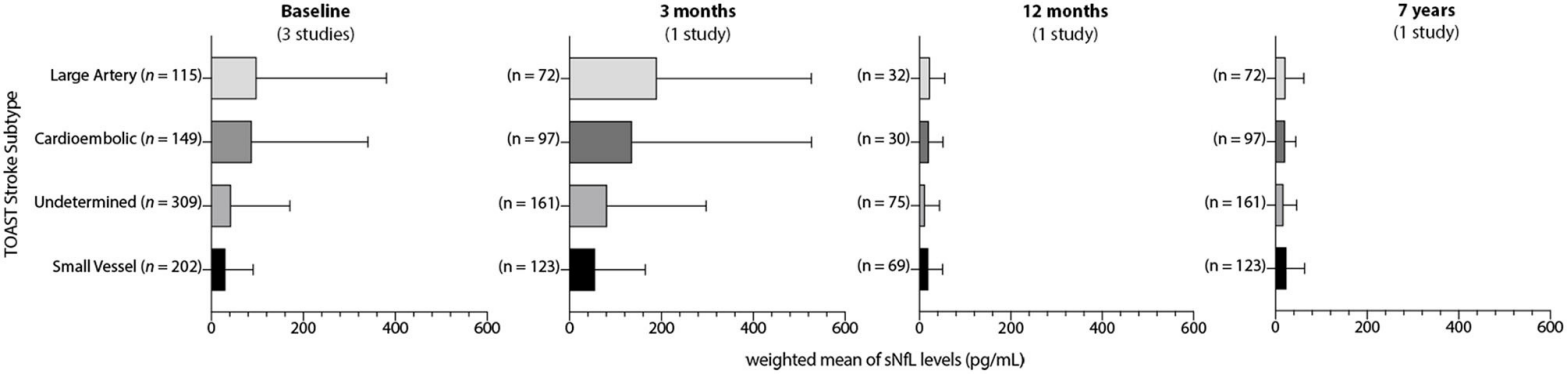
Temporal variance in blood NfL values by acute stroke subtypes. Weighted averages of blood NfL values (pg/mL) by TOAST stroke subtype at available intervals after stroke. Error bars indicate weighted IQR.

Seven articles examined the temporal pattern of blood NfL during the acute, subacute, and chronic phases of injury (12, 15, 17, 20, 21, 27). Though all seven articles assayed blood NfL levels in varying populations and at different time points post-stroke, all seven studies demonstrate a progressive increase in blood NfL peaking in the immediate subacute period between 3 weeks and 3 months after stroke. In a small cohort (*n* = 11), Nielsen et al. did not show a significant increase in serum NfL levels between baseline (<8 h) and 3 days. Gattringer et al. measured serum NfL values in 79 participants with recent small subcortical infarcts (RSSI) in the first 11 days after stroke and in 49 participants at 3 months and 15 months post-stroke. Serum NfL levels increased steadily during the first 11 days, peaked at 3 months [91.34 pg/ml (55.2–127.4)], and then declined to control levels [42.63 pg/ml (26.4–58.9) vs. 34.59 (23.8–45.4)]. In a follow-up study from this cohort, Pinter et al. (13) showed that in subjects with cavitary lacunar stroke lesions, NfL values were increased at 3 months from the baseline measurement [97.9 pg/ml (53.8–142) vs. 85.6 pg/ml (22.3–148.9)] compared to those without cavitation. At 15 months after stroke, serum NfL values dropped to levels comparable to those subjects without cavitary lesion development [46.0 pg/ml (30.2–61.8) vs. 50.1 pg/ml (24.6–75.6)]. Tiedt et al. measured serum NfL levels in 196 AIS subjects from hospital arrival and at days 2, 3, and 7 after stroke as well as at a 6-month post-stroke interval. Serum NfL levels increased from baseline and peaked at day 7 [211.2 pg/ml (104.7–442.6)] and thereafter declined at 6 months. Pujol-Calderón et al. measured CSF and serum NfL levels in 109 and 89 subjects, respectively, after AIS. NfL levels were measured in intervals of 2 days over the first week after stroke as well as at 3 weeks and 3–5 months after stroke. Both serum and CSF NfL values rose steadily over the first week, peaked at 3 weeks after stroke [310 pg/ml (110–600)], and then lowered but remained above control levels in the chronic phase [76 pg/ml (33–210)]. Notably, CSF NfL levels were approximately 50-fold higher than those detected in serum. Pedersen et al. examined NfL variation within the first 2 weeks after stroke onset, at 3 months, and 7 years after stroke in a large cohort of AIS subjects (*n* = 595). Serum NfL values increased steadily during the first 2 weeks and continued to increase at 3 months post stroke while nearly returning to control levels by 7 years. Gendron et al. examined plasma NfL values in 227 AIS patients with varying sampling intervals within the first 20 days after stroke onset. Though this study did not perform serial sampling, compared to subjects with plasma NfL values within 8 days of stroke onset, subjects with plasma NfL values measured between 9 and 20 days after stroke onset were significantly increased with a median value of more than 1,000 pg/ml, ~10 times higher than the earlier group. Finally, in a population of 211 AIS subjects with 73.4% long-term 12-month follow-up, Uphaus et al. examined serum NfL levels within 24 h after admission and at 12 months but while NfL levels varied by stroke severity, aggregate values at these two distant time points were not substantially different [24.2 pg/ml (15.6–42.1) vs. 20.1 pg/ml (17.4–34.1). Notably, only three studies (19–21) reliably reported median NfL values by TOAST stroke subtype (Figure 2). In this limited sample, blood NfL values do vary by etiologic subtype with large artery atherosclerotic stroke showing the highest median NfL values. All TOAST stroke subtypes demonstrate similar temporal variation with an acute elevation, a peak in the subacute period, and a decline but incomplete return to control values in the chronic phase.

### Association of Blood NfL Values With Stroke Outcome

Seven articles specifically addressed the association of admission blood NfL values with stroke outcome data as measured by the modified Rankin Scale (mRS). Four of six studies found an association of blood NfL values with dichotomized stroke outcome measured at 3 months. In a subgroup of 168 AIS patients, Gendron et al. showed that higher baseline plasma NfL was significantly associated with a poor functional outcome defined as a mRS>3 with a hazard ratio of 2.30. Wang et al. had detailed outcome data on all 343 patients from their cohort. Serum NfL values were significantly higher in those with poor functional outcome compared to those with good function outcome (mRS <3) [24.1 pg/ml (18.8–33.9) vs. 15.7 pg/ml (11.9–21.8), respectively]. Uphaus et al. similarly obtained 3- and 36-month outcome data for their entire 211 patient cohort. They showed in both univariate and multivariate analyses that serum NfL could predict AIS patients with a good functional outcome (mRS <2) (OR 1.56). Pedersen et al. tracked serum NfL values longitudinally at 3 months together with outcome data for 532 patients. In this cohort, persistently elevated serum NfL was associated with an OR of 5.83 for a poor functional outcome (mRS>2) after stroke. This effect of sustained elevations of serum NfL at 3 months remained associated with poor functional outcome at 2- and 7-year intervals after stroke (*n* = 320). Onatsu et al. had detailed 3-month outcome data in 89 patients from their cohort. They found that serum NfL correlated with mRS at 3 months but did not find an association with a dichotomized good outcome when controlling for relevant co- factors. Nielson et al. showed a correlation between mRS at 3 months and serum NfL in their restricted cohort. De Marchis et al. did not find an association of serum NfL with mRS outcome at 3 months after stroke. Of the seven available studies that address stroke outcome, measurement of blood NfL values at 3 months rather than during the acute phase appears to provide the strongest predictive value on stroke outcome.

### Blood NfL Values in Other Cerebrovascular Conditions

Our search term strategy also generated five articles that did not examine acute stroke but included populations of individuals with cerebrovascular risk factors including sporadic cerebral small vessel disease (CSVD) and cerebral autosomal dominant arteriopathy with subacute infarcts and leukoencephalopathy (CADASIL). All five studies utilized the SIMOA platform to measure blood NfL levels. Duering et al. (14) measured serum NfL values using the SIMOA platform in 53 CADASIL patients and 439 subjects with sporadic CSVD. These authors demonstrated that serum NfL values were elevated in both sporadic CSVD and CADASIL compared to control [50.8 pg/ml (31.4–70.3) vs. 41.7 pg/ml (18.3–65.2) vs. 26.0 pg/ml (18.7–33.4), respectively]. They also showed that serum NfL was associated with multiple imaging and clinical features of both sporadic CSVD and CADASIL. Peters et al. (26) showed that serum NfL values [53.4 pg/ml (38.1–77.7)] associated not only with baseline white matter lesion burden, mean diffusivity, and cerebral microbleeds but also with incident lacunar infarctions and cognitive deficits in the 503-subject Radboud University Nijmegen Diffusion Tensor and Magnetic Resonance Imaging Cohort (RUN-DMC). Gravesteijn et al. (16) measured serum NfL values in a 41-subject cohort of genetically confirmed *Notch3* mutation-positive CADASIL patients. They showed that baseline serum NfL was significantly increased in patients compared to controls [6.31 pg/ml (2.68–11.67) vs. 1.66 pg/ml (1.25–4.56)] and correlated tightly with MRI and cognitive measures of disease severity. Importantly, these authors demonstrated that baseline serum NfL predicted 7-year cognitive outcomes and long-term survival. Chen et al. (24) used a cohort of 63 CADASIL subjects to monitor disease progression by showing that higher baseline plasma NfL was associated with a nearly 2-fold increased risk of incident stroke. Finally, in a subset of 113 patients from the Action to Control Cardiovascular Risk in Diabetes (ACCORD) trial at risk for stroke who later developed stroke, baseline serum NfL values were predictive of incident stroke and added significant power to the Framingham Stroke Risk Score in this population enriched for the cerebrovascular risk factors of age and diabetes (18).

### Phasic Detection of Blood NfL Levels in Stroke and Cerebrovascular Disease

**Figure 3:**
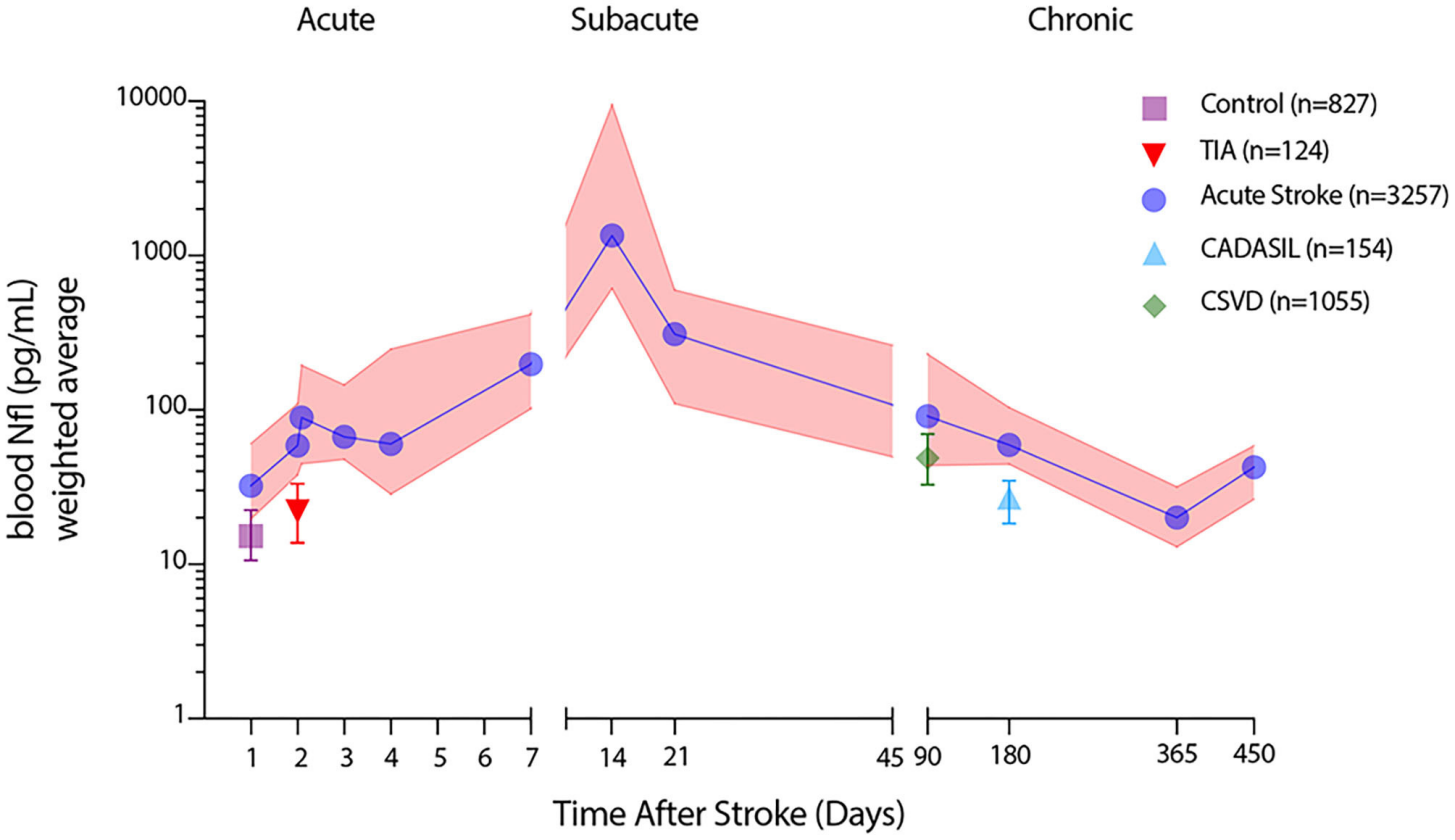
Temporal patterning of blood NfL values measured by SIMOA during distinct acute ischemic stroke epochs. Weighted averages of median blood NfL values (blue) at defined time points after stroke with weighted average interquartile range (pink shading) along with weighted average median blood NfL values and interquartile ranges for healthy controls, transient ischemic attack (TIA), cerebral small vessel disease (CSVD), and cerebral autosomal dominant arteriopathy with subacute infarcts and leukoencephalopathy (CADASIL) subjects.

Given the similarity in immunoassay technique and provision of detailed median and variability data in the majority of human studies identified in this meta-analysis, we sought to create a summary dataset of blood NfL values after stroke. In order to identify reasonable cutoffs that could facilitate the use of blood NfL values in therapeutic clinical trials, we pooled the median and interquartile range raw pg/ml data from the 10 articles that employed the SIMOA platform to measure blood NfL levels in acute ischemic stroke and 5 additional studies examining NfL values in cerebrovascular disease (Table 1). In some instances, primary summary data was available. In other studies when precise timing was not available, we estimated the time range using the median time after stroke for relevant subgroups. Using this data, we created weighted averages and interquartile ranges across the temporal epochs of acute (0–7 days), subacute (9–90 days), and chronic (> 90 days) stroke (Supplemental Data). As comparator groups for acute and chronic stroke time periods, we also included data from studies using the SIMOA platform and examining control groups, TIA subjects, sporadic cerebral small vessel disease, and CADASIL. In total, we included studies measuring blood NfL levels in 2,268 subjects representing 3,257 sampling points after acute stroke. Figure 3 illustrates the temporal variation of weighted average blood NfL values (pg/ml) at acute (0–7 days), subacute (9–90 days), and chronic (>90 days) time intervals after acute stroke presentations with weighted average blood NfL values for relevant comparator populations. In the acute phase, 0–7 days after stroke, mean weighted average blood NfL values were 62.4 pg/ml [IQR 30.2–154.6]. During the subacute phase after stroke 9–90 days after stroke, plasma NfL values spike with a median value of 847.8 pg/ml [IQR 368.7–5201.0] with a peak between 14 and 21 days after stroke. In the chronic phase after stroke, NfL values decline steeply with a median of 73.5 pg/ml [IQR 37.9–173.2] but remain above control and within the range of several chronic cerebrovascular disease values (control = 15.2 pg/ml [IQR 10.6–22.5], *n* = 827; CSVD = 48.7 pg/ml [IQR 32.8–69.6], *n* = 1,055; CADASIL = 27.0 pg/ml [IQR 18.4–34.7], *n* = 154). Excluding AIS, we compared weighted average blood NfL values from these cerebrovascular disease subtypes against each other (Table 2). Blood NfL levels measured on the SIMOA platform can be used to discriminate cerebrovascular disease subtypes (*p* < 0.0001 by one-way ANOVA).

**Table 2:** Comparative blood NfL levels by cerebrovascular disease subtype.

**Cerebrovascular subtype comparisons**			**Absolute difference of weighted average NfL (pg/ml)**	**Adjusted ***p***-value**
TIA	vs.	Control	9.12	0.22
CADASIL	vs.	Control	11.8	<0.0001
CSVD	vs.	Control	33.52	<0.0001
RSSI	vs.	Control	64.2	<0.0001
CADASIL	vs.	TIA	2.7	0.98
CSVD	vs.	TIA	24.4	<0.0001
RSSI	vs.	TIA	55.0	<0.0001
CSVD	vs.	CADASIL	21.7	<0.0001
RSSI	vs.	CADASIL	52.4	<0.0001
RSSI	vs.	CSVD	30.6	<0.0001

*Subjects by cerebrovascular disease subtype: RSSI (n = 65), TIA (n = 44), CSVD (n = 1,055), CADASIL (n = 154), Control (n = 1,055). Significance determined using Tukey's multiple comparison test. TIA, transient ischemic stroke; CADASIL, cerebral autosomal dominant arteriopathy with subacute infarcts and leukoencephalopathy; CSVD, cerebral small vessel disease; RSSI, recent small subcortical infarct*.

### Probabilistic Model of Blood NfL Values in Stroke

To determine the relationship of plasma NfL values between time periods after AIS, we generated a series of random effects models across time periods using the median NfL values and variance from the studies using the SIMOA platform (Table 3). Notably, this modeling paradigm generated mean NfL values across studies that were substantially lower than the weighted averages derived from the median values, yet the temporal patterning is similar. In the acute phase after stroke, mean NfL values were significantly higher than control (36.04 vs. 18.85 pg/ml; *p* < 0.05), though the difference was narrow. In the subacute phase, NfL values further increase and are significantly elevated above control values (87.99 vs. 18.85; *p* < 0.05). In the chronic phase after stroke, the difference between stroke and control subjects was minimal but reached statistical significance (27.27 vs. 18.85; *p* < 0.05). In a probabilistic matrix, a blood NfL value of ~46.0 pg/ml generated a likelihood ratio above 2 indicating reliable detection of a diagnosis of stroke. Similarly, a blood NfL value of 55.7 pg/ml generated a likelihood ratio of 10 for a diagnosis of stroke. Insufficient summary data was available to include TIA in the probabilistic matrix though Onatsu et al. previously proposed an estimated cutoff for stroke vs. TIA at 49.0 pg/ml, which is quite similar to the stroke vs. control cutoff in our predictive model. Notably, Zhao et al. found a lower AIS vs. TIA cut-off of 30.3 pg/ml.

**Table 3:** Random effects mixed models for blood NfL values during stroke epochs.

**Model**	**Time period**	**Estimate**	**Std error (pg/ml)**	***t*-value**	**Significance (*p* <0.05)**
Model 1: Estimated NfL value by time period	Control Acute (0–7 d) Subacute (9–90 d) Chronic (>90 d)	18.85 pg/ml 36.04 87.99 27.27	5.60 5.96 14.30 5.82	3.37 6.05 6.15 4.69	n/a Yes Yes Yes
Model 2: Estimated NfL value relative to control	Control Acute (0–7 d) Subacute (9–90 d) Chronic (>90 d)	18.85 pg/ml 17.18 69.13 8.41	5.60 4.37 13.36 3.23	3.37 3.93 5.18 2.60	n/a Yes Yes Yes
Model 3: Estimated log-transformed NfL value by time-period	Control Acute (0–7 d) Subacute (9–90 d) Chronic (>90 d)	2.70 log NfL pg/ml 3.82 4.45 3.32	0.23 0.21 0.29 0.24	12.01 18.02 15.50 3.73	n/a Yes Yes Yes

We created a similar probabilistic matrix to determine the likelihood of predicting a time period after stroke for any given NfL value. To operationalize this tool, we created an interactive web interface allowing users to input a specific plasma NfL value and determine both the likelihood of a stroke diagnosis and the corresponding likelihood of the approximate time window after stroke. This online tool is available at http://axonbiomarker.org.

## Discussion

In this systemic review and meta-analysis of blood NfL values in human subjects with acute ischemic stroke and chronic cerebrovascular disease, we highlight the emerging evidence that blood levels of NfL carry value as a diagnostic and prognostic biomarker for stroke. Incorporating data from nearly 4,000 subjects with blood NfL values measured on a common, ultrasensitive immunoassay platform, we identify a distinct pattern of temporal variation in blood NfL values that peaks during the subacute period ~2–3 weeks after stroke. Using probabilistic modeling from reported data, we created a searchable web interface that returns the likelihood of stroke and the predicted timing after stroke for any given blood NfL measurement taken on the SIMOA platform. Given the significant contribution of axonal injury during and after stroke, understanding the dynamics of axonal degeneration can inform the management of patients with stroke and cerebrovascular disease.

Blood-based biomarkers are poised to serve an important role in the diagnosis and prognosis of stroke and cerebrovascular disease. They may also fill a critical translational gap by providing novel therapeutic strategies with a measurable surrogate for disease states, target engagement, and therapeutic windows. Blood NfL levels are uniquely positioned to fill this role in stroke but understanding the phasic changes during different stroke epochs and the contribution of underlying cerebrovascular disease states is critical to the deployment of blood NfL as a reliable biomarker. In this study, we sought to identify studies examining circulating levels of neurofilament light chain in research subjects with stroke or cerebrovascular disease states as an example of such a biomarker. Importantly, a comprehensive review of the literature identified no pre-clinical studies with reliable blood NfL data. As blood NfL measurement in clinical populations expands, this creates a significant translational gap that should be filled urgently. Our literature review identified 19 clinical studies that specifically measured blood NfL levels in subjects with stroke or cerebrovascular disease states, including 16 studies that used a common immunoassay platform. Surprisingly, no studies specifically examined the benefit from revascularization treatment on blood NfL levels though most did look at functional outcomes. Despite using only summary level data, we nonetheless were able to generate a clear picture of the temporal dynamics of blood NfL levels across stroke epochs showing a steep rise in the subacute period and a steady but slow decline that is independent of stroke subtype. Further delineation of the effect of stroke subtype, treatment, and baseline risk factors may enhance the probabilistic model and interactive web platform created here.

### Role of Axon Loss in Different Stroke Epochs

As the summated data and models generated in this meta-analysis suggest, the leakage of neurofilament light chain protein into the bloodstream follows a predictable pattern after stroke, rising slowly in the first few days, peaking between 14 and 21 days after stroke onset, and then returning to a new baseline, suggestive of at least three distinct phases of axonal damage after stroke. Notably, our analysis indicates that this pattern is somewhat independent of ischemic stroke subtype or their severity, though larger strokes, particularly those detectable on initial head CT, appear to show markedly higher levels of blood NfL. The limited studies that reported median/IQR values for blood NfL by TOAST subtype suggest that large vessel strokes lead to the largest initial and sustained increase in NfL levels however only a small number of studies report this data.

In the initial acute period after stroke, blood NfL values are variable but based on the summary data collected in this study, they can be relied upon to distinguish stroke from TIA when applying reasonable cutoff thresholds. Broader use of circulating NfL as a diagnostic tool for other acute neurologic diseases is unclear. This paradigm shares a parallel with cardiac troponin in which clinical symptoms of myocardial ischemia provides some diagnostic specificity, but plasma measures of cardiac troponin further increase the sensitivity of detection by reflecting end-organ ischemic tissue damage. While the highly sensitive diffusion-weighted MRI technique is often used to detect ischemic tissue damage, it is still not universally available acutely in many stroke care settings, making the potential contribution of blood-based biomarkers such as NfL, attractive diagnostic tools.

During the subacute phase after stroke when blood NfL values peak, our analysis suggests that this must coincide with a peak in the degradation of the neuronal cytoskeleton and clearance mechanisms must be engaged to remove damaged axons. Though damage to axons occurs within minutes to days after stroke onset (33, 34), the blood-brain barrier demonstrates dynamic changes after stroke (35–37), indicating that the delayed increase in blood NfL detection likely does not reflect sustained opening of the blood-brain barrier but rather slow enzymatic degradation and release of this neuronal cytoskeletal component as well as the potential activation of cerebral clearance mechanisms. This delayed subacute peak in axonal damage perhaps indicates that molecular pathways regulating the breakdown of injured axons (Wallerian degeneration) may be a therapeutic target with direct prognostic implications for recovery. In the chronic phase after stroke, blood NfL values drop precipitously to near control levels but remain persistently elevated above control up to a year after stroke. In this chronic phase, it may be difficult to distinguish those with a prior history of stroke from those with chronic cerebrovascular disease states on blood NfL values alone.

### Utility of Blood NfL as a Clinical Trial Enrollment or Outcome Tool

Because of its potential dual role as both a prognostic biomarker and target engagement biomarker, blood NfL holds particular promise for utility as a criterion for clinical trial enrollment or surrogate outcome measure for trials investigating stroke therapeutics. Analysis of the studies included in this meta-analysis suggest that there is a clear prognostic association between elevated blood NfL and stroke outcome as measured by 90 d mRS. More severe strokes lead to greater axonal injury and therefore worse functional outcomes. This fits well with available neuroimaging data showing that the extent of injury to vital motor fiber tracts such as the corticospinal tract is correlated with functional recovery (5, 38, 39). An obvious detail lacking from blood NfL measures is the fiber tract specificity of axonal injury. A lacunar stroke may densely damage the corticospinal tract and lead to a relatively small bump in blood NfL levels while the primary clinical deficit from a larger middle cerebral artery occlusion may result largely from similar damage to the corticospinal tract, but yet blood NfL values may be expected to be substantially higher in this larger stroke due to axonal injury in other tracts or cortical regions. This discrepancy may also directly influence the relationship between NIHSS scores and NfL values such that lower NIHSS scores more tightly correlated with NfL values while higher NIHSS scores are more likely to show a non-linear relationship with higher variance. Thus, a deeper look at individual temporal trajectories of blood NfL variance in different stroke subtypes are needed in order to precisely utilize this biomarker as a functional tool to track the effectiveness of a stroke therapeutic. The addition of blood NfL measurement at 3 months post-stroke may add additional fidelity in evaluating stroke therapeutic interventions as it appears associated with longer-term functional outcomes. However, if coupled with appropriate neuroimaging techniques such as DTI and tractography, blood NfL values measured serially during post-stroke epochs may be quite valuable in determining the extent of fiber tract damage without challenging quantitative neuroimaging analysis.

While motor recovery after stroke is a key functional metric that drives improvement on the modified Rankin scale, there is increasing awareness that post-stroke cognitive impairment also directly influences functional outcome after stroke (40). The work of Peters et al. suggests that blood NfL does associate with future cognitive impairment and as such, plasma NfL levels are currently being evaluated as a potential biomarker for vascular cognitive impairment in the MarkVCID consortium effort (41). The role of neuro-axonal injury as a driver of post-stroke cognitive impairment is also a central feature under investigation in the recently launched Determinants of Incident Stroke Cognitive Outcomes and Vascular Effects on Recovery (DISCOVERY) Study (42). Thus, understanding the contribution of baseline elevations in blood NfL levels in both chronic cerebrovascular risk and after acute stroke are critical to the application of prognostic biomarkers for VCID and post-stroke cognitive impairment. While our interactive web interface currently addresses only the data from acute stroke studies, we anticipate being able to expand this to include emerging data and a more nuanced interpretation consistent with the principles of precision medicine in stroke (43). For example, blood NfL levels are known to be elevated in other neurologic disease states that damage axons including multiple sclerosis, Alzheimer's disease, and traumatic brain injury (44–46), several of which are commonly co-morbid in individuals with stroke. How these conditions modulate the interpretation of blood NfL values after stroke is presently unknown.

### Limitations

This analysis has several limitations. Though in most cases, we were able to extract median and variance data for specific subgroups, our analysis lacks the detail available with subject level data. This fact may blunt the interpretation of specific blood NfL values and obscures important subgroups that could be stratified by stroke severity or other critical metrics. Secondly, some of the data extracted from the available studies provided time ranges for blood NfL measurement after AIS rather than specific time intervals. As a result, these values needed to be estimated in order to build a probabilistic time-after-stroke model. This limitation particularly plagued the critical early subacute period for which there is the least amount of data. Thirdly, there was a wide variety of variant stroke populations in certain articles ranging from TIA to cerebral small vessel disease to large vessel stroke resulting from various etiologies. Our analysis was therefore agnostic to potentially critically important subject variables including demographic features such as age, sex, co-morbid neurodegenerative disease, and to a lesser degree ischemic stroke subtypes. Notably, plasma NfL levels have been shown to be increased by up to 3-fold in individuals with lower glomerular filtration rates (47), thus correction for renal function will be essential in future studies. Finally, though our model included only studies using a common and robust assay platform (SIMOA), this analysis ignores any differences between serum and plasma measurement of blood NfL levels as well as minor lot variations in reagents that can introduce variability. An individual patient level data meta- analysis incorporating critical co-variates is needed to advance this valuable and accessible translational tool.

## Conclusions

Stroke is a complex disease compounded by patients with prodromal risk conditions, variable etiologies, severities, and trajectories of recovery. These features mandate a precision medicine approach. As shown here, blood levels of NfL measured during and after stroke can carry significant diagnostic and prognostic value. Biomarkers that directly measure brain injury are poised to play a vital role in tailoring aspects of care in the acute, subacute, and chronic phases of stroke and cerebrovascular disease.

## Data Availability Statement

The original contributions presented in the study are included in the article/Supplementary Material, further inquiries can be directed to the corresponding author.

## DEMDAS Study Group

M. Dichgans, M. Endres, S. Wunderlich, I. Zerr, M. Goertler, T. Klockgether, and G.C. Petzold.

## Author Contributions

JS and RM: conceptualization, investigation, data curation, formal analysis, visualization, and writing–original draft. KM: investigation, data curation, formal analysis, visualization, and writing–review and editing. DC: software, data curation, and visualization. IL: conceptualization and writing–review and editing. TU, TW, and ST: data curation, resources, and writing–review and editing. KG: resources, supervision, and writing–review and editing. JH: conceptualization, methodology, formal analysis, visualization, supervision, funding acquisition, and writing–original draft. All authors contributed to the article and approved the submitted version.

## Funding

Funding support was provided by the Lillian R. Gleitsman Medical Student Summer Research Program NIA T35AG026736 (JS), NINDS U19 NS115388 (JH), NINDS U19 NS120384 (JH), and UH3 NS100608 (JH). The DEMDAS study received funding from the German Center for Neurodegenerative Diseases (DZNE), Bonn, Germany.

## Conflict of Interest

JS received funding from NIA T35AG026736. TU reports grants from advanceCOR, grants from Else Kröner-Fresenius Stiftung (2018_EKMS.21), personal fees from Merck Serono, personal fees from Pfizer, outside the submitted work. KG received honoraria for advisory boards or speakers bureau for Abbott Medical, Bayer Vital GmbH, Bristol-Myers Squibb, Portola and Springer Medizin Verlag GmbH outside the submitted work. ST reports receiving funding from the Corona foundation outside of the submitted work. JH reports grants from NINDS NS115388, NS120384, and NS100608 and is an inventor on US Patent 16/487,322, and Founder of Sage Cerebrovascular Diagnostics, Inc outside of the submitted work. The remaining authors declare that the research was conducted in the absence of any commercial or financial relationships that could be construed as a potential conflict of interest.

## Publisher's Note

All claims expressed in this article are solely those of the authors and do not necessarily represent those of their affiliated organizations, or those of the publisher, the editors and the reviewers. Any product that may be evaluated in this article, or claim that may be made by its manufacturer, is not guaranteed or endorsed by the publisher.

## Supplementary Material

Supplemental Data Summary quantitative data used in meta-analysis of blood NfL values in stroke and cerebrovascular disease.

Supplementary Figure 1 Assessment of bias figure.
