# Supplementary figures and images for: Temporal Patterning of Neurofilament Light as a Blood-Based Biomarker for Stroke: A Systematic Review and Meta-Analysis
Source: Front Neurol. 2022 May 16;13:841898. doi: 10.3389/fneur.2022.841898 (PMC9149427; doi:10.3389/fneur.2022.841898)

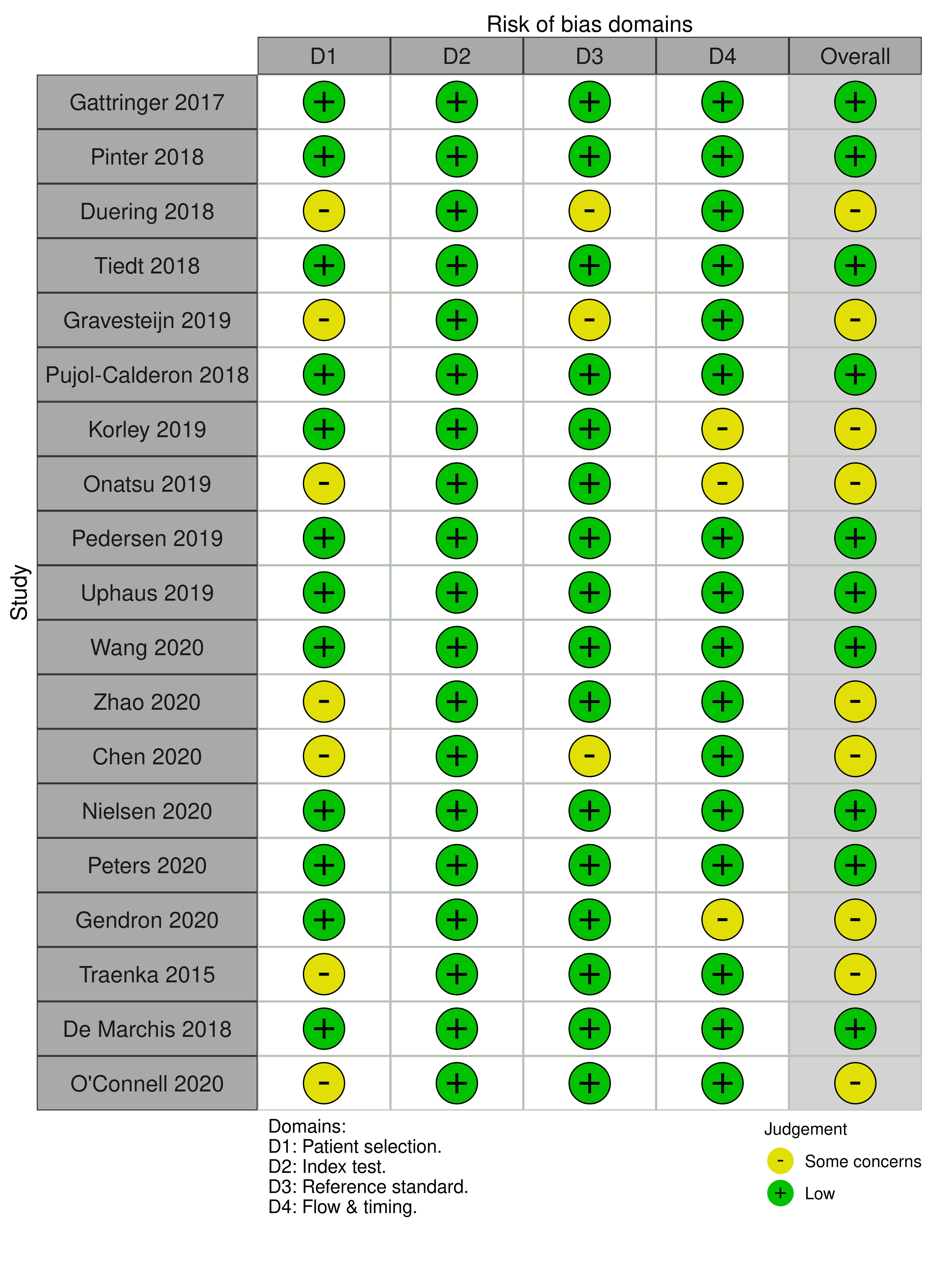

Supplement: Supplementary Figure 1 — Assessment of bias figure. [file Image_1.JPEG]
